# A rare case of oesophageal rupture: Boerhaave's syndrome

**DOI:** 10.1186/s12245-014-0027-2

**Published:** 2014-07-01

**Authors:** Gerben van der Weg, Marald Wikkeling, Maarten van Leeuwen, Ewoud ter Avest

**Affiliations:** 1 Department of Emergency Medicine, Medical Centre Leeuwarden, Henry Dunantweg 2, Leeuwarden 8934, BR, the Netherlands; 2 Department of Surgery, Nij Smellinghe Hospital, Drachten 9202, NN, the Netherlands; 3 Department of Radiology, Nij Smellinghe Hospital, Drachten 9202, NN, the Netherlands

**Keywords:** Boerhaave's syndrome, Oesophageal rupture, Treatment

## Abstract

A 70-year-old patient was referred to our emergency department with severe retrosternal pain after forceful vomiting. Computed tomography (CT) scan revealed a left-sided oesophageal rupture with accompanying pneumomediastinum and bilateral pleural effusions. Conservative treatment with cessation of oral intake, intravenous broad-spectrum antibiotics, parenteral fluids and nutrition and left sided tube thoracostomy was initiated initially. After 5 days, however, the patient deteriorated. Follow-up CT scan demonstrated a mediastinal fluid collection as well as loculated pleural empyema. Open thoracotomy with mediastinal debridement and pleural drainage was performed, after which he made a slow but full recovery. Spontaneous oesophageal rupture due to an abrupt rise in intraluminal pressure caused by vomiting is also known as Boerhaave's syndrome. It is a rare but potentially life-threatening condition. Many patients present with atypical symptoms, and therefore, physicians should have a high index of suspicion in any patient presenting with vomiting and retrosternal pain. When Boerhaave's syndrome is suspected, a CT scan of the thorax and upper abdomen should be performed since treatment depends on clinical and radiological findings. Conservative management (cessation of oral intake, nasogastric decompression, administration of intravenous fluids and parenteral nutrition, intravenous broad-spectrum antibiotics and proton pump inhibitors and tube thoracostomies) may only be considered in patients with a contained rupture without systematic symptoms of infection. In these patients, endoscopic bridging of the tear with a self-expandable stent is also an option. Primary surgical repair (either by thoracotomy or by video assisted thoracoscopy (VATS)) should be considered when patients present with sepsis and/or large non-contained leaks or with severe mediastinal decontamination.

## Background

Spontaneous perforation of the oesophagus after forceful vomiting is also known as Boerhaave's syndrome. It most often occurs in the distal posterolateral aspect of the oesophagus [[1],[2]]. Many patients present with atypical symptoms like shock or respiratory distress, and findings on physical exam are often non-specific, with tachycardia, tachypnea or fever. Not surprisingly, Boerhaave's syndrome is often misdiagnosed as an aortic emergency, pericarditis, myocardial infarction, pulmonary embolus, spontaneous pneumothorax, perforated peptic ulcer or pancreatitis [[3],[4]]. We outline the case of a 70-year-old man, who presented to the ED with retrosternal pain after vomiting, and discuss the clinical presentation, appropriate diagnostic steps and treatment strategies of this rare but potentially-life threatening condition.

## Case presentation

**Figure 1:**
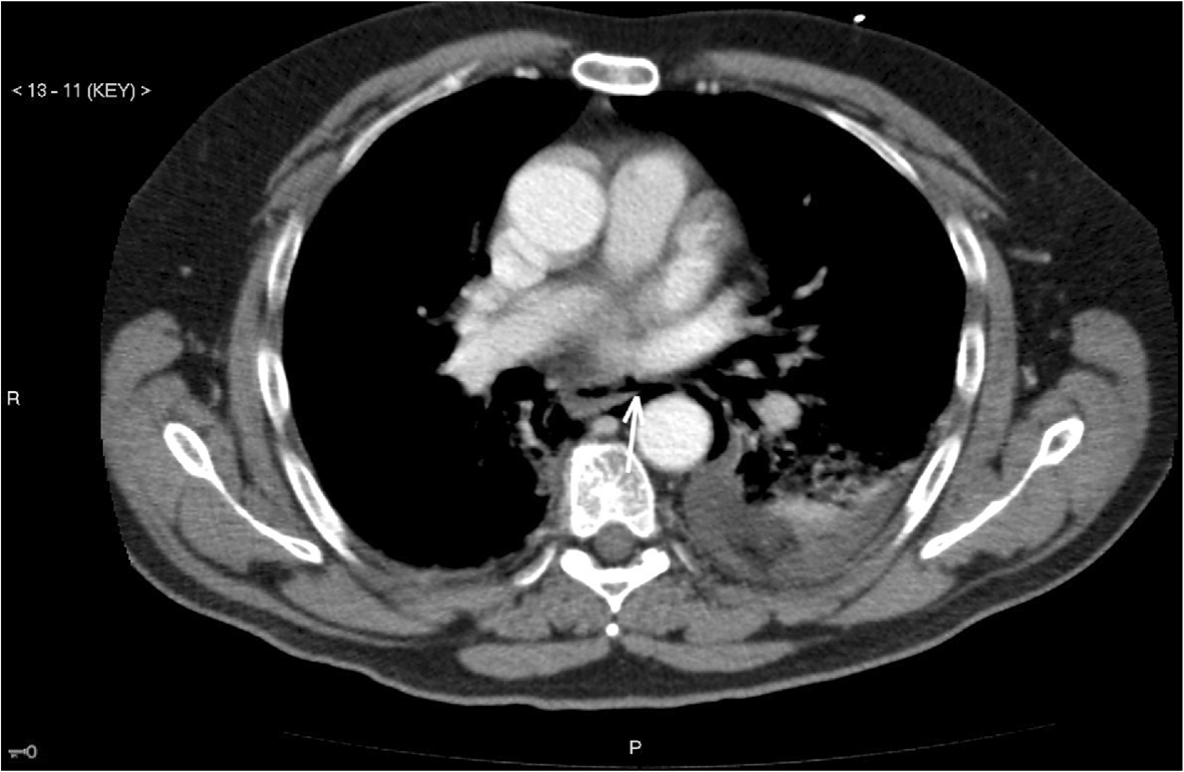
Oesophageal rupture with air leakage into the mediastinum (white arrow) and left sided pleural effusion.

**Figure 2:**
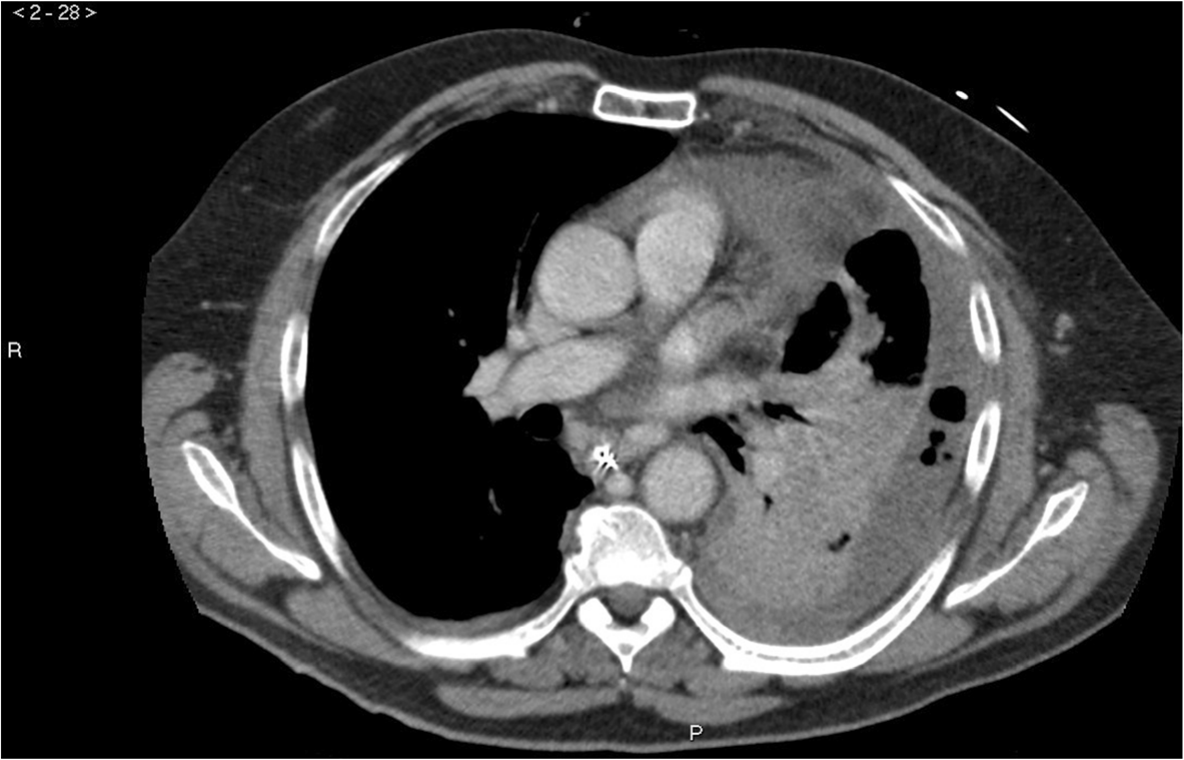
**Complications of the oesophageal rupture.** Mediastinitis (induration of the mediastinal fat) and extensive left-sided pleural effusion with air pockets.

A 70-year-old man with a history of hypertension was referred to our emergency department with a severe retrosternal and upper abdominal pain that started after he had been vomiting several hours before presentation. At admission, he was diaphoretic and in respiratory distress. Blood pressure was 210/100 mmHg, pulse rate 95 beats/min, oxygen saturation was 95% and core temperature was 36.1°C. Physical examination revealed extensive cervical and thoracic subcutaneous emphysema but was otherwise unremarkable. Laboratory results were normal by the time of presentation. A computed tomography (CT) scan revealed a rupture in the left distal part of the oesophagus, a pneumomediastinum and left-sided pleural effusions (Figure 1). Conservative treatment, with cessation of oral intake, nasogastric suction, administration of intravenous fluids and parenteral nutrition, intravenous broad-spectrum antibiotics, proton pump inhibitors and drainage of the pleural effusion by left-sided thoracostomy was initiated in the ICU. After 5 days, however, he developed a fever. Follow-up CT scan demonstrated severe mediastinal contamination and left-sided loculated pleural empyema (Figure 2). Open thoracic surgery was performed with debridement and drainage of the mediastinum and the pleural cavity, after which he made a slow but full recovery.

## Discussion

Many patients with Boerhaave's syndrome present with atypical symptoms like shock or respiratory distress, and findings on physical exam are often non-specific. The classical ‘Macklers triad’ consisting of (repeated) vomiting (79%), lower chest pain (83%) and subcutaneous emphysema (27%) is only present in a minority of the patients. Not surprisingly, it is often misdiagnosed as an aortic emergency, pericarditis, myocardial infarction, pulmonary embolus, spontaneous pneumothorax, perforated peptic ulcer or pancreatitis [[3],[4]].

Further radiological studies should be performed in any patient with a suspicion of Boerhaave's syndrome. Plain chest X-ray is in over 90% of the cases abnormal, with most often mediastinal or free peritoneal air as the initial manifestation [[5]]. Less often, with cervical oesophageal perforations, prevertebral or subcutaneous air may be present. Despite the high prevalence of plain chest X-ray abnormalities, contrast enhanced CT scan of the chest and upper abdomen is the preferred examination. Although it may not always directly localize the site of the perforation, it can detect oesophageal wall oedema, extra-oesophageal air, peri-oesophageal fluid collections and air and fluid in the pleural spaces and retroperitoneum with a higher sensitivity than plain chest X-ray [[6]]. Since CT findings (together with clinical parameters) are used to determine the degree of containment of the rupture and the accessibility of any fluid collections for percutaneous or surgical drainage, they help guide subsequent treatment.

Management of oesophageal perforations can be primarily conservative, endoscopic or surgical. The best treatment approach depends on the extent, location and containment of the perforation and the patients' delay in presentation and comorbidities. Conservative management (cessation of oral intake, nasogastric decompression, administration of intravenous fluids and parenteral nutrition, intravenous broad-spectrum antibiotics, proton pump inhibitors and tube thoracostomies) may be considered in patients with a contained rupture of the oesophagus without mediastinal or pleural contamination on imaging studies and without systemic symptoms of infection at the time of presentation [[7]–[10]]. However, only a minority of patients with Boerhaave's syndrome will fulfil these criteria (as opposed to, e.g. patients with iatrogenic perforations). As a result of forceful vomiting, pleural and/or mediastinal contamination with gastric content is usually present. Primary surgical or endoscopic treatment is therefore warranted in most patients.

Endoscopic (temporary) bridging of the tear with a self-expandable stent is an option in non-septic patients, as well as in patients with comorbidities that preclude surgery. However, although stenting has the potential for early oral feeding and a reduced hospital length of stay, case series published so far are small [[11]–[13]]. In addition, a recent study by Sweigert et al. [[14]] demonstrated that endoscopic stent insertion had a higher mortality than primary operative management. Primarily surgical intervention should take place in all patients with non-contained perforations, when comorbidities that preclude surgery [[9],[15]] are absent. The most successful surgical approach involves primary repair of the oesophagus, especially when the patient presents to the hospital early (within 24 h). Adequate mediastinal debridement can be done by open thoracotomy, or video-assisted thoracoscopy (VATS), with results for VATS being comparable with those for open thoracotomy [[16],[17]]. In the presence of a diseased oesophagus, resection may be the appropriate treatment. No consensus exists whether patients can be managed with primary surgical repair when there is a significant delay in presentation or diagnosis, although a study carried out in a more heterogeneous group of patients with oesophageal ruptures could not demonstrate a relation between mortality risk and delay in presentation [[15]]. In retrospection, our patient might have benefitted from primary, instead of delayed surgery, since a pleural effusion was already present on the initial CT scan.

## Conclusions

Our case clearly demonstrates the importance for any physician to have a high index of suspicion for Boerhaave's syndrome in any patient presenting with vomiting and retrosternal pain, and it stresses the importance of early aggressive (surgical) treatment.

## Consent

Written informed consent was obtained from the patient for publication of this case report and any accompanying images. A copy of the written consent is available for review by the editor-in-chief of this journal.

## Competing interests

The authors declare that they have no competing interests.

## Authors' contributions

GvdW and EtA drafted the manuscript. GvdW, MW and MvL were directly involved in patient care and revised the manuscript critically. All authors read and approved the final manuscript.
